# Endometrial stromal PRMT5 plays a crucial role in decidualization by regulating NF-κB signaling in endometriosis

**DOI:** 10.1038/s41420-022-01196-x

**Published:** 2022-10-04

**Authors:** Xinyu Cai, Manlin Xu, Hui Zhang, Mei Zhang, Junxia Wang, Jie Mei, Yang Zhang, Jidong Zhou, Xin Zhen, Nannan Kang, Qiuling Yue, Haixiang Sun, Ruiwei Jiang, Guijun Yan

**Affiliations:** 1 Center for Reproductive Medicine and Obstetrics and Gynecology, Nanjing Drum Tower Hospital, Nanjing University Medical School, Nanjing, China; 2 Center for Molecular Reproductive Medicine, Nanjing University, Nanjing, China; 3 State Key Laboratory of Reproductive Medicine, Nanjing Medical University, Nanjing, China; 4 State Key Laboratory of Pharmaceutical Biotechnology, Nanjing University, 210032 Nanjing, China

**Keywords:** Infertility, Mechanisms of disease

## Abstract

Decidualization is a prerequisite for successful embryo implantation, in which elongated fibroblast-like endometrial stromal cells differentiate into more rounded decidual cells. Accumulating evidence has stressed the important role of the defective eutopic endometrium in infertility in endometriosis patients. However, the role of arginine methylation in the process of physiological decidualization and pathological decidualization defects is not clear. Here, we observed that the expression level of PRMT5, the main type II PRMT, was decreased in the endometrium of endometriosis patients, predominantly in stromal cells. Compared with the undecidualized state, PRMT5 was increased in the stromal cells of normal secretory endometrium in humans and in the decidua of normal pregnant mice or mice with artificially induced decidualization. The inhibition of PRMT5 resulted in a significant decrease in uterine weight and decidualization-related regulator expression, including FOXO1, HOXA10 and WNT4, in mice and IGFBP1 and prolactin levels in human endometrial stromal cells. Transcriptome analysis showed that decreased PRMT5 activity led to NF-κB signaling activation by inducing p65 translocation to the nucleus, which was also observed in endometriosis patients. Finally, overexpression of PRMT5 rescued the defective expression of IGFBP1 and prolactin in primary endometrial stromal cells from endometriosis patients. Our results indicate that promotion of PRMT5 may provide novel therapeutic strategies for the treatment of decidualization defects in infertile women, such as those with endometriosis.

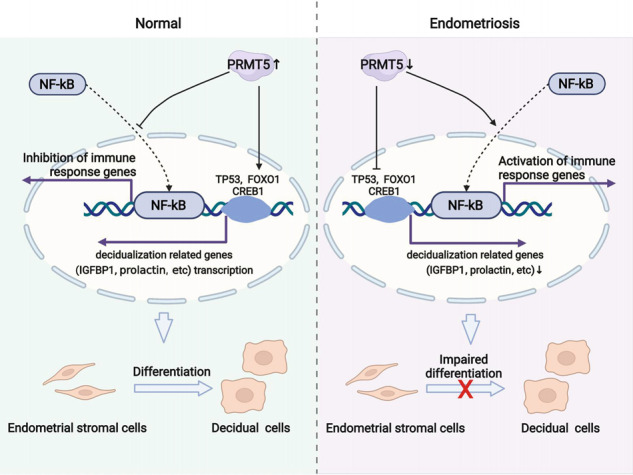

## Introduction

Endometriosis is a reproductive disorder in which endometrial tissue is aberrantly located outside the uterus. Almost 10% of reproductive-age women suffer from endometriosis related infertility and pelvic pain [1]. Women with endometriosis are two times more likely to experience infertility and pregnancy loss compared with those without endometriosis [2, 3]. Accumulating evidence has stressed the important role of the defective eutopic endometrium in infertility in endometriosis patients. A previous study found that the prevalence of endometriosis was 47% in those infertile women with regular cycle whose partners have a normal semen analysis [4]. However, even in patients with donated oocyte treatment, the implantation rate and clinical pregnancy rate were both reduced in those endometriosis patients than that in the women without endometriosis receiving sibling oocytes from the same donor [5]. Induction of endometriosis in animals has been shown to lead to embryo implantation failure due to a defective decidualization response, which is similar to the pathological phenotype found in endometriosis patients [6, 7].

Decidualization occurs during the secretory phase of the menstrual cycle controlled by progesterone and other ovarian hormones, which is a prerequisite for successful embryo implantation [8]. Decidualizing cells undergo changes in cell morphology from elongated fibroblast-like endometrial stromal cells towards a rounded or polygonal shaped decidual cells, in which a large amount of secretory proteins were produced, including prolactin (PRL) and insulin-like growth factor binding protein-1 (IGFBP1), two established decidualization markers [9]. Molecular and genetic evidence has indicated multiple regulatory pathways by which decidualization defects might arise, and many have been identified as aberrant in endometriosis, including estrogen, progesterone, forkhead box O1 (FOXO1), AKT, and Notch pathways [10–13]. In addition, some researches have found that the endometriotic tissue presented different expression patterns of DNA methylation compared with normal endometrium, and decreased expression of homeobox A10 (HOXA10) and progesterone receptor (PR) due to markedly elevated methylation at the promoter has been proven to contribute to the defective decidualization in the endometrium of endometriosis patients and induced endometriosis animals [14–17]. However, the pathophysiological significance of the posttranslational modification regulatory machinery, such as arginine methylation, in endometriosis is still not clear.

Protein arginine methyltransferases (PRMTs) are responsible for arginine methylation by transferring methyl groups from S-adenosylmethionine to a guanidine nitrogen of arginine in proteins, to generate three types of methylated arginine residues: monomethylarginine (MMA), asymmetric dimethylarginine (aDMA), and symmetric dimethylarginine (sDMA). PRMTs have been proven to be implicated in the regulation of many biological processes, including development and cancer [18]. Depending on catalytic activity, PRMTs can be classified as a type I enzyme that catalyzes aDMA (PRMT1, PRMT2, PRMT3, PRMT4, PRMT6 and PRMT8) or a type II enzyme that generates sDMA (PRMT5, PRMT7 and PRMT9) [19]. A recent study found that aDMA content and PRMT3 expression were increased in the decidua of recurrent miscarriage patients, primarily in macrophages but not in natural killer cells or stromal cells of the decidua [20]. However, the localization and function of PRMT5, the main type II PRMT, is not clear in the endometrium. Here, we found that PRMT5 expression was decreased in the mid-secretory endometrium of endometriosis patients, predominantly in stromal cells. Inhibition of the expression and activity of PRMT5 blunted endometrial decidualization in mice. Inhibition of PRMT5 activity impaired human endometrial stromal cell decidualization partly by activating the NF-kappa B (NF-κB) signaling pathway, while overexpression of PRMT5 rescued the decidualization defect in endometrial stromal cells from endometriosis patients.

## Results

### Decreased PRMT5 expression in the endometrial stromal cells of endometriosis patients

**Fig. 1:**
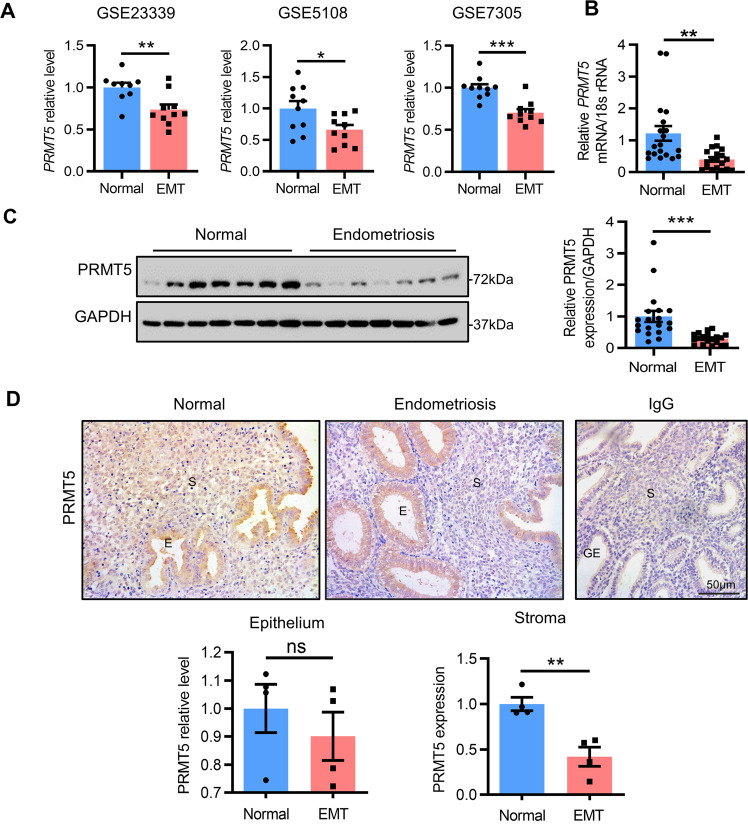
Decreased expression of PRMT5 in the endometrial stromal cells of endometriosis patients. **A** The mRNA levels of PRMT5 in the ectopic endometrium from women with endometriosis (EMT) and endometrium of healthy controls from GSE23339, GSE5108 and GSE7305. **B** qRT–PCR analysis and **C** Western blot analysis of PRMT5 in the mid-secretory phase eutopic endometrium from women with (EMT: *n* = 19) or without (normal: *n* = 19) endometriosis. **D** IHC analysis of PRMT5 in the mid-secretory phase eutopic endometrium from women with (EMT: *n* = 4) or without (normal: *n* = 4) endometriosis. Scale bar = 50 µm; E epithelium, S stroma. Means ± SEM. ^*^P < 0.05, ^**^P < 0.01, ^***^P < 0.001, Student’s *t*-test.

We first screened the expression levels of *PRMT5* mRNA from several published gene expression profiles of endometriosis [21–23] and observed that the relative expression levels of *PRMT5* were significantly decreased in the ectopic endometrium of endometriosis patients compared to the endometrium of healthy controls (Fig. 1A). We next determined *PRMT5* mRNA expression in the mid-secretory phase eutopic endometrium of endometriosis patients. Our qPCR showed that the expression level of *PRMT5*, but not *PRMT1* or *PRMT3*, was obviously decreased in the eutopic endometrium of endometriosis patients compared with that in fertile controls (Fig. 1B, Supplementary Fig. 1A, B). Western blot data further confirmed the decreased level of eutopic endometrial PRMT5 in endometriosis patients (Fig. 1C). Immunohistochemical staining (IHC) analysis revealed that the reduction in PRMT5 expression in the eutopic endometrium of endometriosis patients was observed mainly in stromal cells but not in epithelial cells (Fig. 1D). The above results indicated that PRMT5 was decreased in the eutopic endometrium of endometriosis patients, especially in stromal cells, prompting us to explore the potential role of endometrial stromal PRMT5.

### Endometrial stromal PRMT5 expression increases upon decidualization in mice

**Fig. 2:**
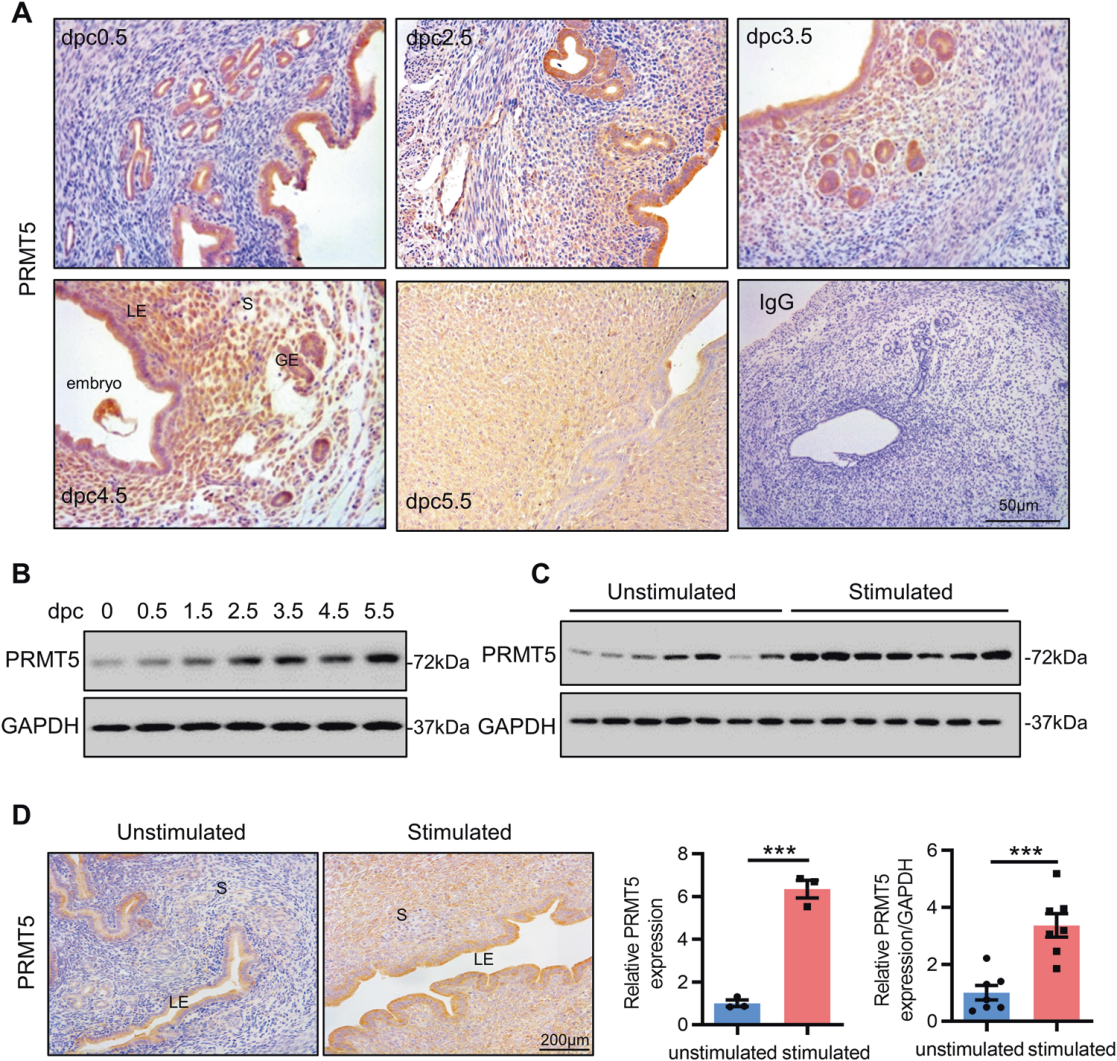
Endometrial stromal PRMT5 expression increases upon decidualization in mice. **A** IHC analysis of PRMT5 in the uterus at 0.5–5.5 days postcoitum (dpc) of pregnancy, scale bar = 50 µm. **B** Western blot analysis of PRMT5 expression in the uterus at 0.5–5.5 dpc of pregnancy. **C** Western blot analysis of PRMT5 expression in the stimulated and unstimulated uteri in an artificially stimulated decidualization mouse model (*n* = 7). **D** IHC analysis of PRMT5 in the stimulated and unstimulated uteri in an artificially stimulated decidualization mouse model (*n* = 3), scale bar = 200 µm. LE luminal epithelium, GE, glandular epithelium, S stroma. Means ± SEM. ^***^P < 0.001, Student’s *t*-test.

To address the pathophysiological significance of PRMT5 in endometrial stromal cells, we first analyzed the PRMT5 expression pattern in the peri-implantation mouse uterus by IHC. PRMT5 was primarily expressed in luminal epithelial cells at 0.5 days postcoitum (dpc) but expanded to uterine luminal and glandular epithelial cells and stromal cells at 3.5 dpc when the uteri entered receptive status [24]. With the onset of embryo implantation at 4.5 dpc, PRMT5 was detected in both epithelial and stromal cells surrounding the implanting blastocysts and became more visible in the decidualizing cells on 5.5 dpc (Fig. 2A, B). We next assessed the PRMT5 expression pattern in a mouse model of artificially stimulated decidualization. PRMT5 expression increased after decidualization was stimulated, especially in decidualizing cells (Fig. 2C, D). All the above data indicated that PRMT5 may be involved in the process of endometrial decidualization.

### PRMT5 is required for endometrial decidualization in mice

**Fig. 3:**
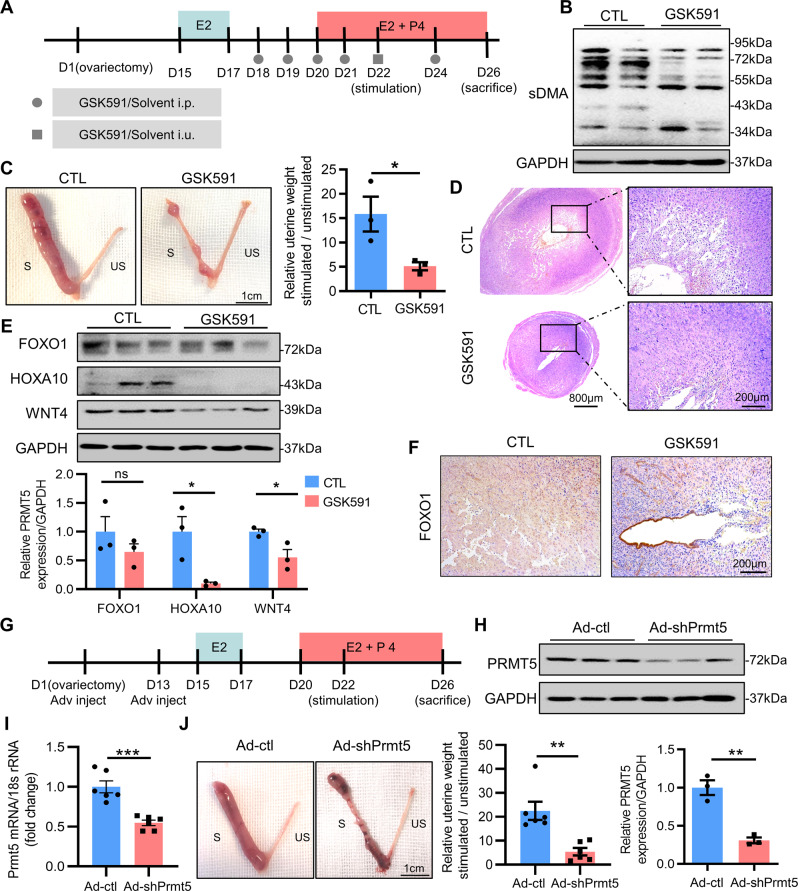
PRMT5 is required for endometrial decidualization in mice. **A** Schematic representation of stimulated decidualization procedures with GSK591 treatment. **B** Western blot analysis of SDMA level in the stimulated uteri from the control and GSK591 groups. **C** Gross morphology of the unstimulated or stimulated uterine side and the ratio of stimulated to unstimulated uterine weight from the control (CTL) and GSK591 groups, scale bar = 1 cm. **D** Hematoxylin-eosin staining of the stimulated uteri from the control and GSK591 groups, scale bars = 800 µm (left panel) and 200 µm (right panel). **E** Western blot analysis of FOXO1, HOXA10 and WNT4 expression in the stimulated uteri from the control and GSK591 groups. **F** IHC assay of FOXO1 in the stimulated uterus from the control and GSK591 groups, scale bar = 200 µm. **G** Schematic representation of stimulated decidualization procedures with treatment by an adenovirus harboring sh-PRMT5. **H** Western blot analysis of PRMT5 expression in the stimulated uteri from the control (Ad-ctl) and Ad-shPRMT5 groups. **I** qRT–PCR analysis of PRMT5 mRNA in the stimulated uteri from the Ad-ctl and Ad-shPRMT5 groups. **J** Gross morphology of unstimulated or stimulated uterine side and the ratio of stimulated to unstimulated uterine weight from the Ad-ctl and Ad-shPRMT5 groups, scale bar = 1 cm. i.p. intraperitoneal injection, i.u. intrauterine injection, S stimulated, US unstimulated. ^*^P < 0.05, ^**^P < 0.01, Student’s *t*-test.

To clarify the role of PRMT5 in endometrial decidualization, we applied GSK591, a specific inhibitor of PRMT5 [25], to an artificial decidualization mouse model (Fig. 3A, B). After 4 days of oil stimulation, we observed impaired decidualization and decreased decidual tissue weight following GSK591 treatment (Fig. 3C, D). We also assessed the expression of FOXO1, HOXA10 and WNT4, well-known markers for uterine stromal differentiation during decidualization [9]. GSK591-treated mice showed decreased expression of FOXO1, HOXA10 and wingless-type mouse mammary tumor virus integration site 4 (WNT4) after 4 days of oil stimulation compared with the control groups (Fig. 3E). The IHC assay further showed that FOXO1 protein level was obviously suppressed in the stromal cells in the endometrium of GSK591-treated mice compared with that in the decidualizing stromal cells in the decidua of the control mice, although FOXO1 remained in endometrial epithelial cells of GSK591-treated mice (Fig. 3F). In addition, we knocked down PRMT5 in mice following artificially induced decidualization (Fig. 3 G–I) and observed that knockdown of PRMT5 led to impaired decidualization and decreased decidual tissue weight (Fig. 3J). These results suggested that the expression and activity of PRMT5 were indispensable to endometrial decidualization in mice.

### PRMT5 activity ensures human endometrial stromal cell decidualization

**Fig. 4:**
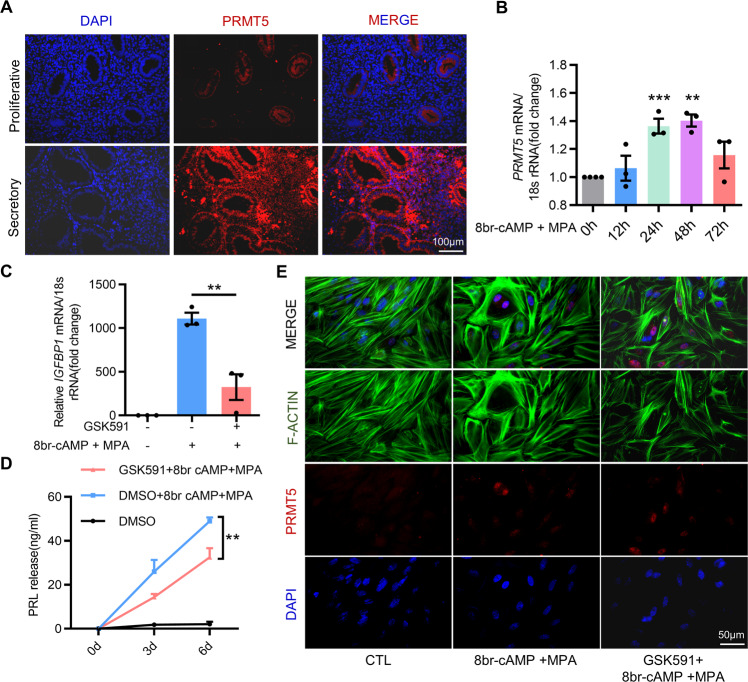
PRMT5 activity ensures human endometrial stromal cell decidualization. **A** Immunofluorescence staining of PRMT5 proteins in the proliferative and secretory phase endometria, scale bar = 100 µm. **B** qRT–PCR analysis of PRMT5 mRNA in human endometrial stromal cells (hEnSCs) treated with medroxyprogesterone acetate (MPA) and 8Br-cAMP for 0, 12, 24, 48 and 72 h. **C** qRT–PCR analysis of IGFBP1 mRNA in hEnSCs treated with or without the PRMT5 inhibitor GSK591 for 24 h before 3 days of treatment with 8Br-cAMP and MPA. **D** ELISA of PRL levels in supernatant obtained from hEnSCs decidualized for 3 and 6 days with or without the PRMT5 inhibitor GSK591. **E** Immunofluorescence staining for F-actin in hEnSCs treated with or without the PRMT5 inhibitor GSK591 before 3 days of treatment with 8Br-cAMP and MPA, scale bar = 50 µm. Means ± SEM. ^**^P < 0.01, ^***^P < 0.001, One-way ANOVA with Tukey’s multiple comparisons test in **B**, Student’s *t*-test in **C**, Two-way ANOVA with Tukey’s correction for multiple comparisons in **D**.

We next assessed the significance of PRMT5 on the decidualization of human endometrial stromal cells (hEnSCs). Immunofluorescence staining showed that PRMT5 was predominantly localized in the epithelial cells of the proliferative endometrium, while its localization was expanded to epithelial and stromal cells in the secretory endometrium (Fig. 4A). The expression of PRMT5 increased markedly upon decidualization with 8-bromo-cAMP and medroxyprogesterone acetate (8Br-cAMP+MPA) (Fig. 4B). Treatment with GSK591 obviously blocked the increased IGFBP1 expression and PRL secretion, two important decidual marker genes [9], in 8Br-cAMP+MPA-treated hEnSCs (Fig. 4C, D). We also examined whether inhibition of PRMT5 affects cytoskeletal organization. As shown in Fig. 4E, decidualized hEnSCs displayed polygonal cell morphologies and an increased number of nuclei in the expanding cytoplasm, but GSK591 treatment impeded the transformation from a long fibroblast-like shape into a round shape in hEnSCs. These findings indicated the significant role of PRMT5 activity in human endometrial stromal cell decidualization.

### Inhibiting PRMT5 activates the NF-κB signaling pathway in human endometrial stromal cells

**Fig. 5:**
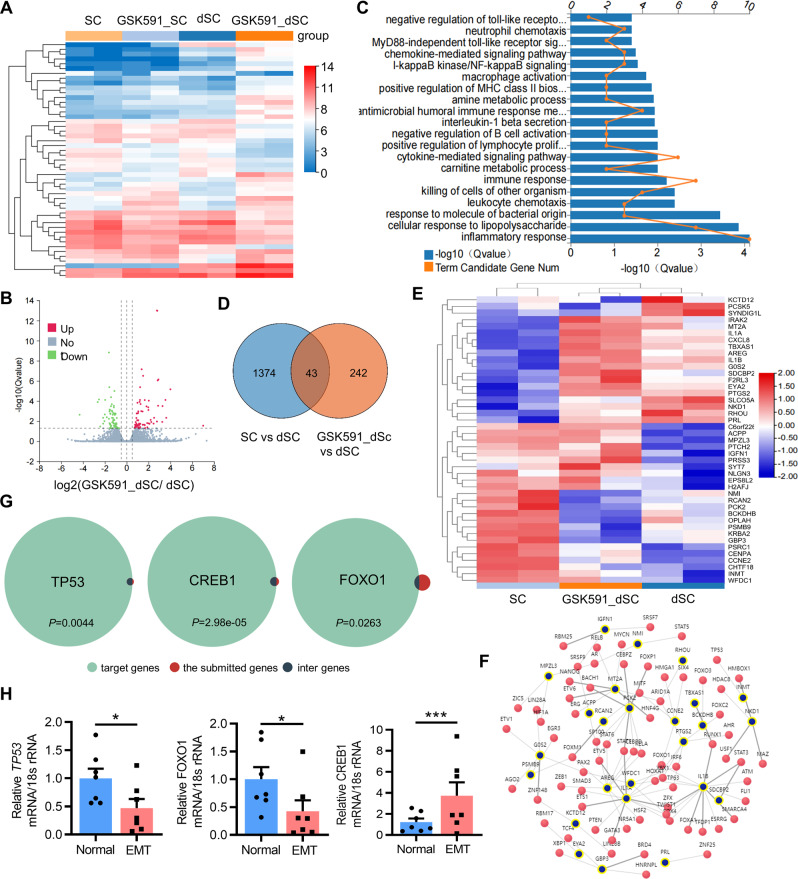
Analysis of transcriptome alterations in PRMT5 activity-inhibited hEnSCs. **A** Heatmap of differentially expressed genes between GSK591-treated and solvent-treated hEnSCs (SC) or decidualized hEnSCs (dSC). **B** Volcano plot of significantly downregulated (green dots) and upregulated (red dots) genes in PRMT5 activity-inhibited dSC (GSK591_dSC) compared with dSC. **C** GO biological process enrichment analysis of differentially expressed genes between GSK591_dSC and dSC. **D** Venn diagram showing the overlap of differentially expressed genes among the two comparisons, **E** and heatmap of the 43 overlapping genes. **F** Subnetwork analysis of transcription factors and their target genes in the 43 differentially expressed genes associated with decidualization. **G** Transcription factor enrichment of the 43 differentially expressed genes associated with decidualization. **H** qRT–PCR analysis of the mRNA levels of TP53, FOXO1 and CREB1 in the mid-secretory phase eutopic endometrium from women with (EMT: *n* = 7) or without (normal: *n* = 7) endometriosis. ^*^P < 0.05, ^***^P < 0.001, Student’s *t*-test.

To further determine the role of PRMT5 in decidualization, RNA-seq analysis was performed on control hEnSCs, GSK591-treated hEnSCs, decidualized hEnSCs and GSK591-treated decidualized hEnSCs (Fig. 5A). There were 137 genes upregulated and 148 genes downregulated in GSK591-treated decidualized hEnSCs compared with decidualized hEnSCs (Fig. 5B). Gene ontology (GO) enrichment analysis of genes that showed changes between GSK591-treated decidualized hEnSCs and decidualized hEnSCs showed abundant enrichment in immune- and inflammatory-related pathways (Fig. 5C). We next explored the decidualizing-related genes regulated by PRMT5. Forty-three out of 1374 changed genes between decidualized hEnSCs and control hEnSCs were observed in the 242 changed genes between GSK591-treated decidualized hEnSCs and decidualized hEnSCs (Fig. 5D, E). The intersection analysis of the 43 differentially expressed genes and the target genes of multiple transcription factors, including TP53, cAMP response element-binding protein (CREB1) and FOXO1, which have been shown to be closely related to endometrial function and decidualization [9], was conducted (Fig. 5F). Among the 43 differentially expressed genes, there were 37 target genes of TP53, 33 target genes of CREB1, and 11 target genes of FOXO1 (Fig. 5G). Endometriosis patients showed decreased mRNA levels of *TP53* and *FOXO1*, while increased mRNA level of *CREB1* in the mid-secretory phase eutopic endometrium compared with the fertile controls (Fig. 5H).

**Fig. 6:**
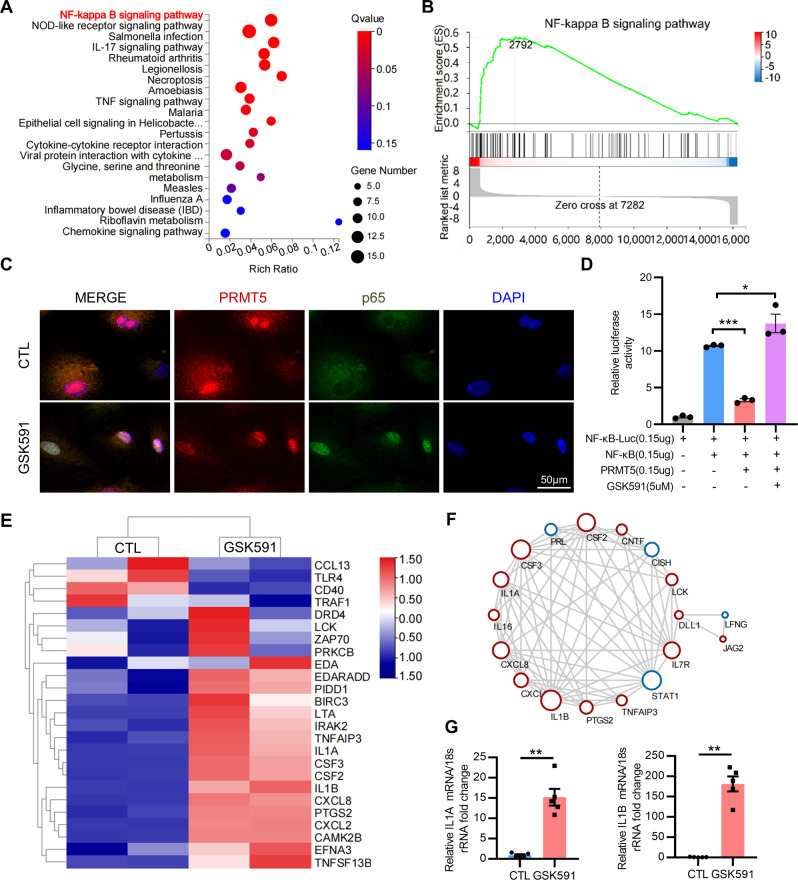
Inhibiting PRMT5 activates the NF-κB signaling pathway in human endometrial stromal cells. **A** The top 20 pathways enriched in differentially expressed genes between decidualized hEnSCs (dSC) and GSK591-treated decidualized hEnSCs (GSK591_dSC) in Kyoto Encyclopedia of Genes and Genomes analysis. **B** The Gene Set Enrichment Analysis revealed a significant enrichment of the NF-κB signaling pathway in GSK591_dSC. **C** Analysis of the immunofluorescence staining of p65 and PRMT5 in hEnSCs treated with or without GSK591 after 72 h of treatment with 8Br-cAMP and MPA, scale bar = 50 µm. **D** Dual-luciferase assays of hEnSCs transfected with NF-κB-luc and Rinella after treated with GSK591, or transfected with pCMV-NF-κB or pCMV-PRMT5 for 48 h. **E** Heatmap of differentially expressed genes associated with the NF-κB signaling pathway. **E** Protein–protein interaction network based on STRING analysis of the genes shown in **D**. **F** qRT–PCR analysis of IL1A and IL1B mRNA in GSK591_dSC and dSC. Means ± SEM, ^*^P < 0.05, ^**^p < 0.01, ^***^P < 0.001, One-way ANOVA with Tukey’s multiple comparisons test in **D**, Student’s *t*-test in **G**.

Kyoto Encyclopedia of Genes and Genomes (KEGG) analysis and Gene Set Enrichment Analysis (GSEA) further showed an enrichment of the NF-κB signaling pathway in GSK591-treated decidualized hEnSCs compared with decidualized hEnSCs (Fig. 6A, B). Immunofluorescence staining revealed the translocation of the NF-κB subunit p65 into the nucleus in GSK591-treated decidualized hEnSCs, suggesting the activation of the NF-κB signaling pathway (Fig. 6C). Luciferase reporter assays revealed that overexpression of PRMT5 inhibited, while treatment of GSK591 promoted the transcriptional activity of NF-κB (Fig. 6D). A cluster heatmap showed the differential expression of NF-κB signaling pathway-related molecules in GSK591-treated decidualized hEnSCs (Fig. 6E). Protein–protein interaction network analysis of differentially expressed genes showed a number of inflammatory factors in GSK591-treated decidualized hEnSCs (Fig. 6F). qRT–PCR data confirmed that the mRNA levels of interleukin 1-α (*IL1A*) and interleukin 1-β (*IL1B*) were increased significantly in GSK591-treated decidualized hEnSCs (Fig. 6G), which was also observed in the endometrium of endometriosis patients (Supplementary Fig. 2). All the above results suggested that activation of the NF-κB signaling pathway may contribute to the decidualization defect in hEnSCs with inhibition of PRMT5 activity.

### PRMT5 rescues decidualization defects in endometrial stromal cells from endometriosis patients

**Fig. 7:**
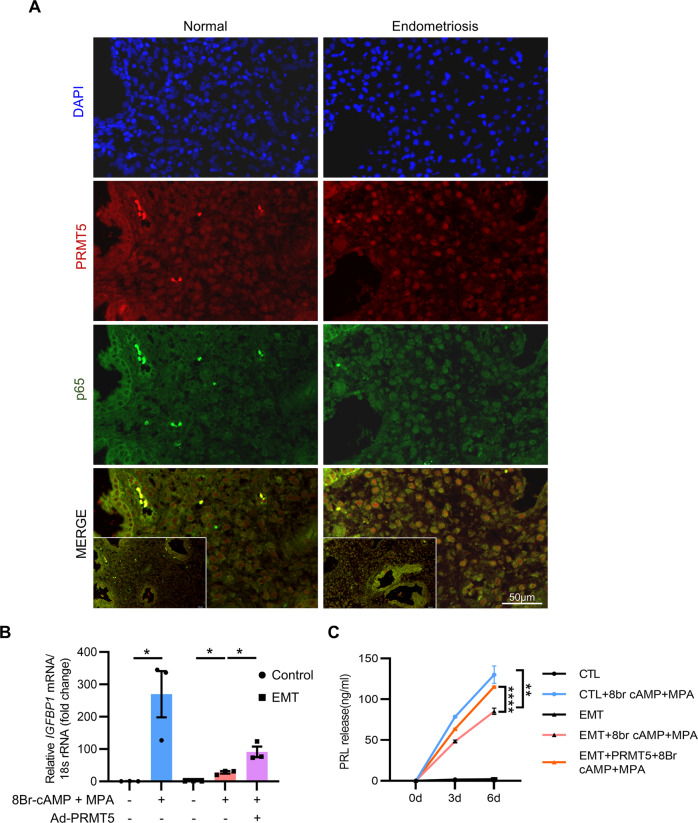
PRMT5 rescues decidualization defects in endometrial stromal cells from endometriosis patients. **A** Analysis of the immunofluorescence staining of p65 and PRMT5 in mid-secretory phase eutopic endometrium from women with or without endometriosis, scale bar = 50 µm. **B** qRT–PCR analysis of IGFBP1 mRNA in hEnSCs from endometriosis patients infected with Ad-PRMT5 for 24 h before 3 days of treatment with 8Br-cAMP and MPA. **C** ELISA results of PRL levels in the supernatant obtained from hEnSCs of endometriosis patients decidualized for 3 and 6 days followed by infection with Ad-PRMT5. Means ± SEM. ^*^P < 0.05, ^**^P < 0.01, ^****^P < 0.0001, One-way ANOVA with Tukey’s multiple comparisons test in **B**, Two-way ANOVA with Tukey’s correction for multiple comparisons in **C**.

Due to the potential regulatory effect of PRMT5 on decidualization, we speculated that overexpression of PRMT5 may improve the decidualization of endometrial stromal cells from endometriosis patients. As expected, p65 was predominantly localized in the nucleus of stromal cells in the mid-secretory phase eutopic endometrium of endometriosis patients (Fig. 7A). Primary hEnSCs isolated from the endometrial tissues of endometriosis patients showed impaired decidualization after differentiation stimulus, as revealed by *IGFBP1* mRNA expression and secreted PRL levels. However, PRMT5 supplementation obviously promoted *IGFBP1* mRNA expression (31.56 vs. 91.18, *P* < 0.05) and secreted PRL levels (85.17 vs. 115.33 ng/mL, *P* < 0.001) (Fig. 7B, C), indicating that PRMT5 rescued the decidualization defect of endometrial stromal cells from endometriosis patients.

## Discussion

Decidualization defects are responsible for impaired endometrial receptivity and embryo implantation failure, contributing to reproductive disorders, such as endometriosis, recurrent implantation failure and recurrent pregnancy loss [26–28]. Arginine methylation mediated by PRMTs has been shown to be an important posttranslational mechanism involved in various biological processes [29]. However, the role of arginine methylation in the process of physiological decidualization and pathological decidualization defects is not clear.

Recently, some studies have reported increased expression of PRMT1 and PRMT3, two type I enzymes, in the endometrium of LPS-induced endometritis rats and the decidua of recurrent miscarriage patients, respectively [20, 30]. By screening the expression levels of various *PRMT* mRNAs from several published gene expression profiles of endometriosis (GSE23339, GSE5108 and GSE7305), we observed that *PRMT5*, a major type II enzyme, was decreased in the ectopic endometrium of endometriosis patients compared to the eutopic endometrium of healthy controls. In humans, circulating progesterone levels are increased following ovulation and maintained at elevated levels during the secretory phase, which is responsible for the decidualization of hEnSCs in nonpregnant cycles [31]. Our data confirmed that the expression of PRMT5 was suppressed in the eutopic mid-secretory endometrium of endometriosis patients, especially in stromal cells. As expected, we observed an obvious increase in PRMT5 in the normal mid-secretory human endometrium and in vitro induced decidualized hEnSCs compared with normal proliferative human endometrium and undifferentiated hEnSCs, respectively. In mice, decidualization does not occur in the normal estrus cycle but is initiated by embryo attachment in normal pregnancy or elicited in hormone-induced mice with intrauterine injection of oil [32]. PRMT5 was observed in the endometrial stromal cells surrounding the implanting blastocyst at 4.5 dpc and became more visible in the decidualizing cells at 5.5 dpc. Elevated PRMT5 during decidualization was further confirmed in a mouse model of artificially stimulated decidualization. GSK591 is a substrate-competitive inhibitor of PRMT5 [25]. We found that GSK591 blunted decidualization in mice with artificially stimulated decidualization and hEnSCs with in vitro stimulated decidualization, which clearly showed that stromal PRMT5 played a crucial role in endometrial decidualization. Furthermore, we confirmed that overexpression of PRMT5 rescued the decidualization defect of primary hEnSCs from endometriosis patients. The human study is limited by its retrospective nature, and a larger sample size or a prospective randomized design could be used in future studies to corroborate the potential effect of PRMT5 on decidualization defects observed in many uterine disorders.

NF-κB is a family of transcription factors composed of homodimers or heterodimers of related Rel proteins, including RelA (p65), p105/p50, p100/p52, c-Rel, and RelB [33]. The upstream stimulus activates the IκB kinase (IKK) complex and restrains inactive NF-κB in the cytoplasm, leading to the translocation of NF-κB to the nucleus and binding to NF-κB DNA consensus sequences in the nucleus, to activate downstream target genes [34]. The functional prediction of the PRMT5 activity-regulated transcriptome via GO analysis, KEGG analysis and GSEA showed that the “NF-κB signaling pathway” was enriched in GSK591-treated decidualized hEnSCs, which was further supported by the translocation of p65 into the nucleus and increased inflammatory factors. NF-κB mediates signaling between IL1A and tumor necrosis factor α (TNFα) and the expression of LIF and IL-6 in endometrial epithelial cells [35], but the detailed regulatory mechanism of NF-κB underlying decidualization is still not clear. A previous study reported that proinflammatory signaling to NF-κB may be suppressed in early pregnancy, contributing to the immunosuppressive mechanism during embryo implantation [36]. While acute inflammation is required for implantation, chronic inflammation has been proven to be disruptive for pregnancy [6]. Endometriosis is characterized by chronic inflammation, which is responsible for the chronic pain, fibrosis and infertility [27]. Some previous studies have shown that the NF-κB signaling pathway was activated in the eutopic secretory endometrium of endometriosis patients, which was also observed in our study, indicating that the absence of decreased p65 activity in the secretory endometrium could participate in endometrial biologic alterations during the implantation window in endometriosis patients [37–39]. In addition, NF-κB inactivation by progesterone and synthetic progestin attenuated the expression of IL-8 in endometriotic stromal cells to control the growth associated with endometriosis [40, 41].

In addition to being regulated by IKK, various posttranslational modifications have been found to be involved in the regulation of NF-κB, including ubiquitination, phosphorylation, acetylation, sumoylation, nitrosylation and methylation [42–46]. Previous studies have showed that p65 is activated by PRMT5 via symmetric dimethylation of arginine 30 and suppressed by PRMT1 via asymmetric dimethylation of arginine 30, indicating the antagonistic relationship between PRMT1 and PRMT5 in regulating NF-κB [44, 46]. Here, we found that inhibiting PRMT5 activity in hEnSCs led to p65 nuclear translocation and NF-κB signaling pathway activation. In addition, p65 nuclear translocation and increased expression of NF-κB signaling-related inflammatory factors were also observed in primary hEnSCs from endometriosis patients with reduced expression levels of PRMT5. However, the detailed mechanism by which PRMT5 symmetrically demethylates p65 to inhibit NF-κB signaling in hEnSCs needs to be further explored.

In summary, we identified PRMT5 as a critical regulator in decidualization both in mice and humans, partly due to its effect on the NF-κB signaling pathway. Since several PRMT5 inhibitors are in ongoing preclinical and clinical studies for the treatment of cancer [47], it is worth observing potential side effects on the endometrium and reproductive capacity. In addition, we confirmed that overexpression of PRMT5 rescued the decidualization defect of primary hEnSCs from endometriosis patients, suggesting that promotion of PRMT5 may provide novel therapeutic strategies for the treatment of decidualization defects in infertile women, such as those with endometriosis.

## Methods and materials

### Sample collection

Endometrial biopsy samples were taken from women undergoing treatment at the Reproductive Medicine Center of Nanjing Drum Tower Hospital (2013-408 081-01). Informed consent was signed by the patients before sample collection. The women in the study were aged between 20 and 40, and none of them had received hormone therapy for at least 3 months prior to tissue collection. The endometriosis group was comprised of women with endometriosis ovarian cysts diagnosed by ultrasound, elevated serum CA125, or endometriosis ovarian cysts confirmed by laparoscopic surgery. The normal group was comprised of women receiving assisted reproductive treatment because of male infertility factors. Mid-secretory endometria timed 6–8 days after ovulation in the natural cycle, as determined by ultrasonography, were obtained from 26 normal women and 26 women with endometriosis for qRT–PCR, western blotting and immunohistochemical staining. Proliferative endometria were obtained from three normal women for immunofluorescence staining. Freshly collected endometrial tissues from 11 normal women and three endometriosis patients were cut into fragments of ~1 mm^3^, digested with 0.1% collagenase, centrifuged and filtered through a screen to obtain the primary human endometrial stromal cells (hEnSCs) for in vitro decidualization experiments as described previously [48]. Detailed information on the participants in this study is summarized in Supplementary Table 1.

### Animals and artificially induced in vivo decidualization

ICR (Institute of Cancer Research) mice (*n* = 32) were purchased from the Lab Animal Center of Yangzhou University (Yangzhou, China). Fourteen mice were subjected to a normal pregnancy assay. To induce in vivo decidualization, 6–8-wk-old female mice were ovariectomized (*n* = 25) with appropriate analgesics. After 14 days, the mice were injected with 100 ng E2 (Sigma-Aldrich, St Louis, MO, USA, #E2758) subcutaneously for three consecutive days. After 2 days of rest, the mice were injected with 1 mg P4 (Sigma-Aldrich, #P0130) and 10 ng E2 subcutaneously for three consecutive days. After the last hormone injection, artificial decidualization was induced in one uterine horn by injection of 20 μL of sesame oil into the lumen. The other uterine horn was left unstimulated as a control. Daily hormone treatments with 1 mg P4 and 10 ng E2 were continued for another 5 days. During this process, the mice were treated with GSK591 (*n* = 3) (MedChem Express, Monmouth Junction, NJ, USA, #HY-100235), Ad-shPrmt5 (*n* = 6) and vehicle (*n* = 9) at times outlined in the procedure in Fig. 3. Six hours after the last hormone injection, the mice were sacrificed, the wet weight of both uterine horns of each mouse was recorded, and uterine tissue was collected and fixed in 4% (wt/vol) paraformaldehyde for histological and immunohistochemical analyses. All animal experiments were approved by the Institutional Animal Care and Use Committee of Nanjing Drum Tower Hospital (No.20210510).

### Construction of adenoviruses

An adenovirus harboring PRMT5 (Ad-PRMT5) was generated using AdMax (Microbix Biosystems, Inc., Toronto, ON, Canada) as previously described [49]. The primers used for Ad-shPrmt5 construction were purchased from Sangon Biotech (Shanghai, China) and designed to target the following cDNA sequence: GCACAGTTTGAGATGCCTTAT (Supplementary Table 2). Then, shPrmt5 was cloned into pacAd5 U6-GFP, followed by adenovirus construction. The adenoviruses were propagated in HEK293A cells and purified via CsCl_2_ banding, followed by dialysis against 20 mmol/l Tris-buffered saline with 10% glycerol.

### Cell culture and in vitro decidualization of human endometrial stromal cells

The primary endometrial stromal cells were isolated and cultured as described previously [48]. Before inducing decidual differentiation, the cells were treated with 5 μM GSK591 (MedChem Express, #HY-100235) or solvent for 24 h and then incubated in DMEM/F12 without phenol red containing 2.5% carbon-adsorbed serum. Medroxyprogesterone acetate (MPA) (1 μM) (Sigma-Aldrich, #71589) and 8-Bromo-cAMP (8-Br-cAMP) (0.5 mM) (Sigma-Aldrich, #B7880) were added to the cellular supernatant. After 48 h-72 h, the decidualization of stromal cells was assessed by evaluating decidualization marker gene expression and cell morphological analysis. For the rescue assay, cells were pretreated with Ad-PRMT5 for 24 h and then treated with 8-Br-cAMP and MPA for 3 days and 6 days.

### Immunohistochemical staining

The tissues were fixed in 4% paraformaldehyde and embedded in paraffin wax. After deparaffinization and rehydration, antigen unmasking was performed by heating the sections in 10 mM sodium citrate buffer (pH 6.0) at 95 °C for 10 min. The sections were then incubated with 3% H_2_O_2_ in deionized water for 15 min to block endogenous peroxidase activity, and nonspecific binding sites were blocked with 10% normal goat serum for 30 min at room temperature. The sections were then incubated with primary antibodies (Supplementary Table 4) in a humid chamber overnight at 4 °C, followed by incubation with the corresponding biotinylated secondary antibody at 37 °C for 30 min. Next, the sections were stained with 3,3′diaminobenzidine (DAB) and counterstained with hematoxylin. Digital images were captured using a Leica DM 2000 microscope and LAS Core software (Leica Microsystems Limited, Wetzlar, Germany). Quantitative analysis of the relative protein expression levels in the epithelial cells and stromal cells of the endometrium samples were determined according to the integrated optical density (IOD) of the digital images (×400) using Image-Pro Plus System 6.0 (Media Cybernetics, Inc., Silver Spring, MD, USA) in a blinded fashion as described previously [50].

### Immunofluorescence staining for PRMT5 and F-actin

hEnSCs were fixed with 4% paraformaldehyde for 20 min at RT and then permeabilized with 0.1% TritonX-100 in PBS for 5 min at RT. Nonspecific sites were blocked with 1% BSA in PBS for 1 h at 37 °C. Endogenous proteins were stained with primary antibodies (Supplementary Table 4) at 4 °C overnight. Fluorescence-conjugated secondary antibodies were used to visualize the signal. Nuclei were stained with 4′,6-diamidino-2-phenylindole dihydrochloride (DAPI) for 10 min. Finally, images were visualized by fluorescence confocal microscopy and processed using ImageJ software.

### Enzyme-linked immunosorbent assay (ELISA)

After 72 h of decidualization, hEnSC culture supernatants were harvested and centrifuged to remove cell debris. PRL levels were measured using a commercially available enzyme-linked immunosorbent assay kit (R&D Systems, Minneapolis, MN, USA, #DPRL00) in collected supernatants according to the manufacturer’s instructions. Samples were assayed in duplicate, and the concentrations were expressed as ng/mL cell supernatant.

### Western blot analysis

Protein was extracted from the tissues or cells using RIPA buffer with a phosphatase inhibitor (Sigma-Aldrich, #P5726) and protease inhibitors (Roche, Branford, CT, USA, #11697498001). The supernatant was extracted after high-speed centrifugation at 4 °C. The protein concentration was determined using the BCA Protein Assay kit (Beyotime, Jiangsu, China, #P0011). Equal amounts of protein (20 μg) were separated by SDS–PAGE and transferred to polyvinylidene difluoride membranes (Merck Millipore, Darmstadt, Germany, #03010040001). The membranes were blocked with 5% skimmed milk for 1 h and then incubated overnight at 4 °C with primary antibodies (Supplementary Table 4). After incubation for 1 h with HRP-conjugated secondary antibodies, detection was performed using an enhanced chemiluminescence kit (Merck Millipore, #32106). The expression of each protein was normalized to the expression of GAPDH in the corresponding sample, and the relative abundance of the target proteins was estimated by densitometric quantification of the signal intensities using ImageJ software.

### RNA isolation and quantitative real-time PCR

Total RNA was extracted from cells using TRIzol (Takara Bio, Shiga, Japan, #T9108) following the manufacturer’s instructions. RNA purity was assessed by measuring the OD at 260 nm and 280 nm, and RNA integrity was assessed by agarose gel electrophoresis. The first strand of DNA (cDNA) was synthesized from total RNA (1 μg) using the Takara PrimeScriptTM RT reagent kit (Takara Bio, #RR037A). The expression levels of genes were analyzed by real-time PCR with SYBR Premix Ex Taq kits (Takara Bio, #RR820A) using appropriate primers (Supplementary Table 3). Relative gene expression levels were calculated with the 2-∆∆Ct method, with 18 S RNA used as the internal control.

### RNA-seq and data analysis

Primary endometrial stromal cells were cultured in 60 mm dishes and treated with GSK591 (MedChem Express, #HY-100235) or solvent for 24 h and then decidualized for another 48 h. Total RNA was extracted from cells using TRIzol (Takara Bio, #T9108). RNA-seq and data analysis were performed by OE Biotech Co., Ltd. (Shanghai, China). Clean reads were mapped to the human reference genome using HISAT2. The DESeqR software package was used to identify DEGs (the threshold was set as *P*-value < 0.05 and |log2foldChange | > 1 for significant difference). ClusterProfiler49 was used to perform GO, KEGG and GSEA analyses. The transcription factor regulatory network was analyzed by KnockTF (http://www.licpathway.net/KnockTF/index.php).

### Dual-luciferase reporter assay

Preconfluent (60%) hEnSCs in 24-well plates were transfected with the indicated plasmids using Lipofectamine 3000 Reagent (Invitrogen, Carlsbad, California, USA, #L3000008). NF-κB-Luc was purchased from Beyotime Biotech (#D2206). Dual-Luciferase Assay System (Promega, Madison, WI, USA, #E2940) was used to analyze the luciferase activities with a luminescence counter (Berthold Technologies) according to the manufacturer’s instructions. Firefly luciferase activity was normalized to the corresponding *Renilla* luciferase activity.

### Statistical analyses

GraphPad Prism 9.0 was used for data statistics and analysis. No statistical methods were used to predetermine the sample size. Mice were randomly allocated to experimental groups. No blinding method was used for animal studies. There was no animal exclusion criteria. The variance was similar between the groups that were being statistically compared. A Student’s *t*-test was used to compare data between two groups, and one-way ANOVA with Tukey’s multiple comparisons was used to compare data from more than two groups. Two-way ANOVA with Tukey’s correction for multiple comparisons was applied for data from two groups with three time points. Data quantification was expressed as the mean ± standard error (X ± SEM), and *P* < 0.05 indicated a significant difference in all cases. Ns = not significant.

## Supplementary information

Supplementary Figures and Figure legends

Supplementary Table 1. Clinical characteristics of women recruited in the study

Supplementary Table 2. Primers used for Ad-shPrmt5 (5'-3')

Supplementary Table 3. Primers used for qRT-PCR (5'-3')

Supplemental Table 4. Details of antibodies used

Original Data File

## Data Availability

The authors provide detailed description of methods and original data upon request. RNA-seq data sets generated in this study have been deposited at the Gene Expression Omnibus (GEO) database under accession number GSE20588.
