# Supplementary material for: Endometrial stromal PRMT5 plays a crucial role in decidualization by regulating NF-κB signaling in endometriosis
Source: Cell Death Discov. 2022 Oct 4;8:408. doi: 10.1038/s41420-022-01196-x (PMC9532444; doi:10.1038/s41420-022-01196-x)
Supplement: Supplementary file 1 — Supplementary Figures and Figure legends [file 41420_2022_1196_MOESM1_ESM.pdf]

Supplementary Figures

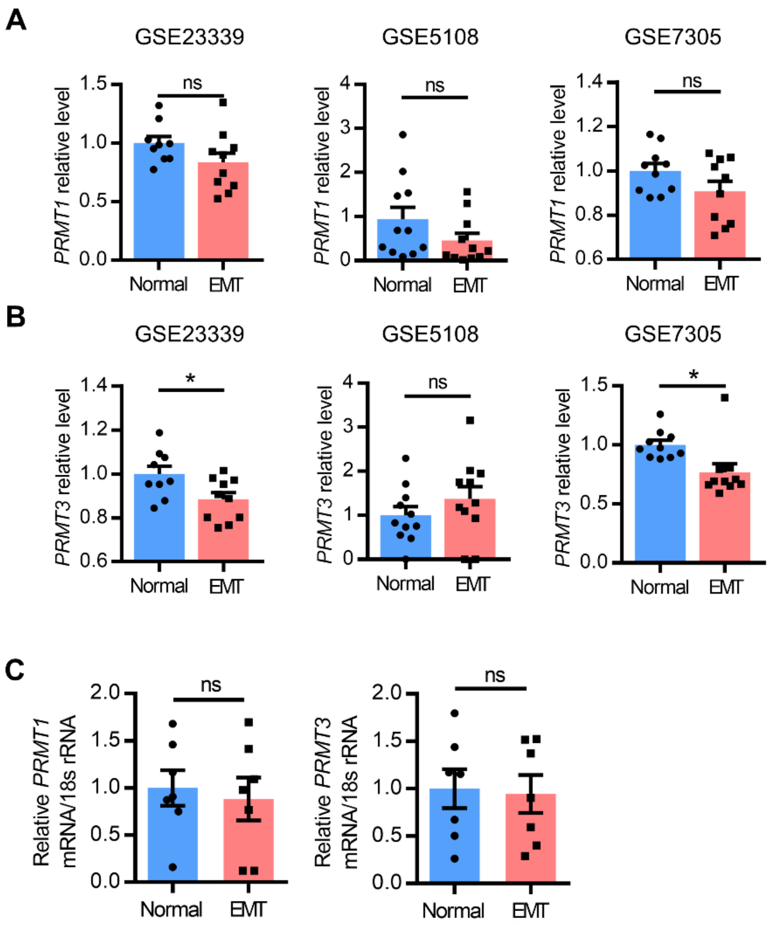

Supplementary Figure 1. Expression of PRMT1 and PRMT3 in endometriosis patients. (A) The mRNA levels of PRMT1 and PRMT3 in the ectopic endometrium from women with endometriosis (EMT) and endometrium of healthy controls from GSE23339, GSE5108 and GSE7305. (B) qRT-PCR analysis of PRMT1 and PRMT3 mRNA in the mid-secretory phase eutopic endometrium from women with (EMT: n=7) or without (Normal: n=7) endometriosis. Means ± SEM. $^{*}P < 0.05$; ns, not significant; Student's t test.

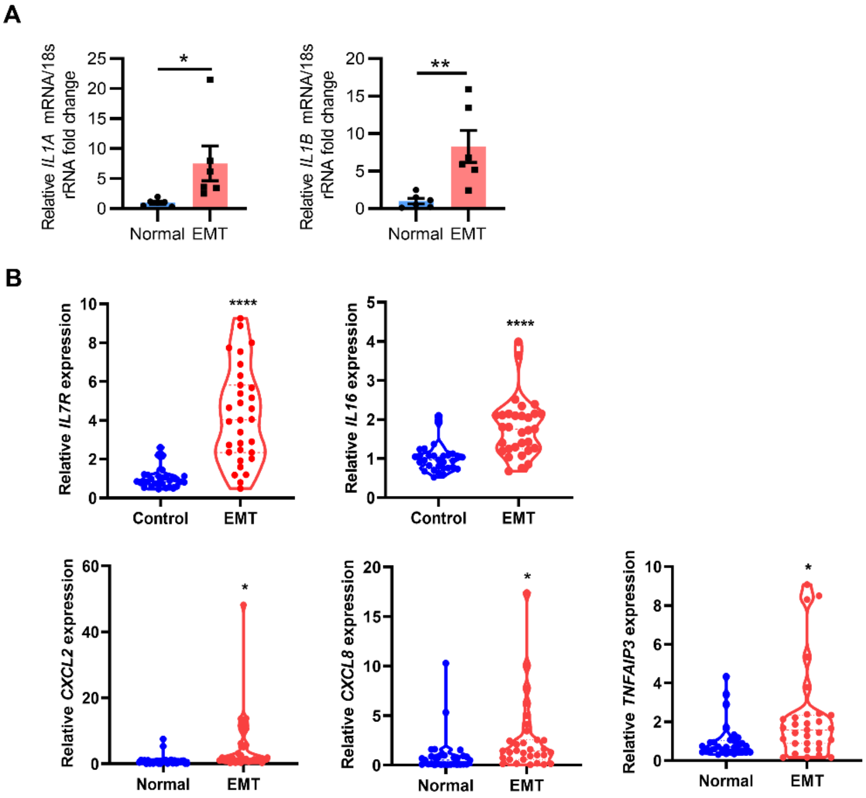

Supplementary Figure 2. Increased inflammation in endometriosis patients..

(A) qRT-PCR analysis of IL1A and IL1B mRNA in the mid-secretory phase eutopic endometrium from women with (EMT: n=7) or without (Normal: n=7) endometriosis. Means ± SEM. $^{*}P < 0.05$; $^{**}P < 0.05$; Student's t test. (B) The relative mRNA levels of IL7R, IL16, CXCL2, CXCL8 and TNFAIP3 in the ectopic endometrium from women with endometriosis (EMT) and endometrium of healthy controls from GSE23339, GSE5108 and GSE7305. Means ± SEM. $^{*}P < 0.05$, $^{**}P < 0.01$, $^{***}P < 0.001$, Student's t test.
