# Supplementary material for: Endometrial stromal PRMT5 plays a crucial role in decidualization by regulating NF-κB signaling in endometriosis
Source: Cell Death Discov. 2022 Oct 4;8:408. doi: 10.1038/s41420-022-01196-x (PMC9532444; doi:10.1038/s41420-022-01196-x)
Supplement: Supplementary file 6 — Original Data File [file 41420_2022_1196_MOESM6_ESM.pdf]

Figure1

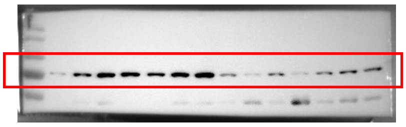

Figure1C_PRMT5

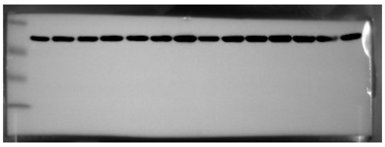

Figure1C_GAPDH

Figure2

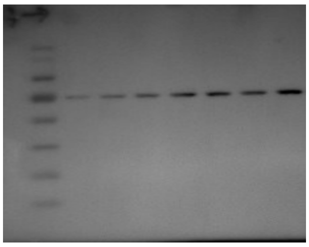

Figure2B_PRMT5

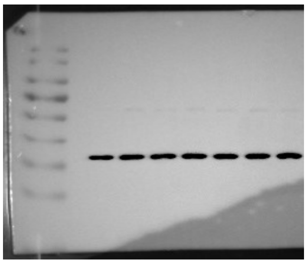

Figure2B_GAPDH

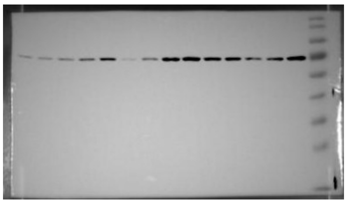

Figure2C_PRMT5

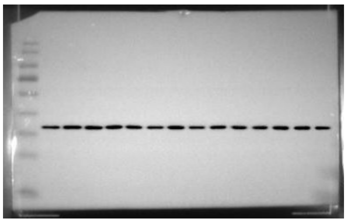

Figure2C_GAPDH

Figure3

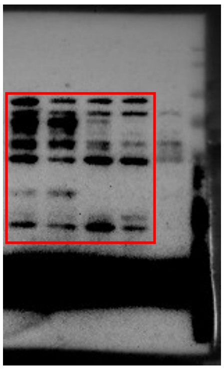

Figure3B_SDMA

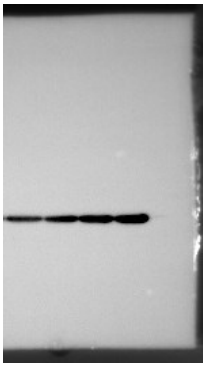

Figure3B_GAPDH

Figure3

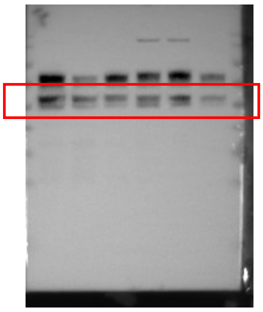

Figure3E_FOXO1

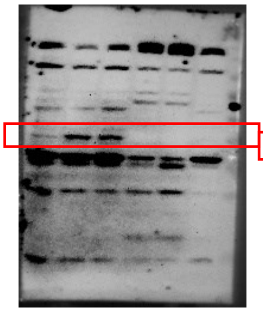

Figure3E_HOXA10

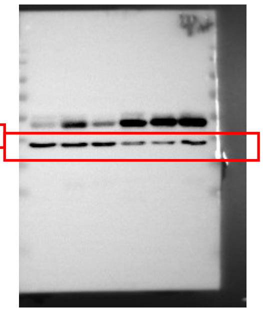

Figure3E_WNT4

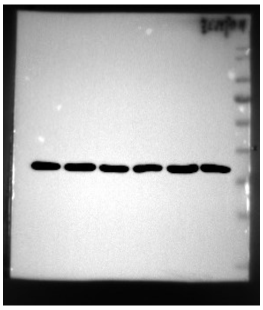

Figure3E_GAPDH

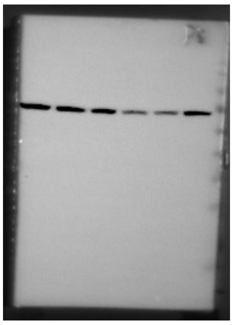

Figure3H_PRMT5

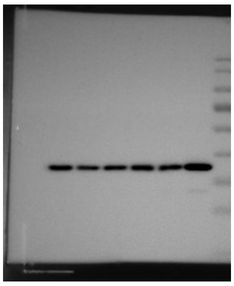

Figure3H_GAPDH
